# Not all masses are metastases: a diagnostic dilemma resolved – synchronous HER2-positive breast cancer and renal oncocytoma

**DOI:** 10.1097/RC9.0000000000000459

**Published:** 2026-04-09

**Authors:** Neelesh Shrivastava, Saikat Mitra, Shantanu Pande

**Affiliations:** a Department of Surgical Oncology, AIIMS, Nagpur, Maharashtra, India; b Department of Pathology, AIIMS, Nagpur, Maharashtra, India; c Department of Nuclear Medicine, AIIMS, Nagpur, Maharashtra, India

**Keywords:** breast neoplasm, case report, multidisciplinary care, multiple primary neoplasms, partial nephrectomy, positron emission tomography computed tomography, renal oncocytoma

## Abstract

**Introduction:**

In oncology, the discovery of a synchronous renal mass during breast cancer staging often signifies metastatic disease, drastically altering prognosis and management. We present a critical case where this assumption was challenged by a rare benign entity, renal oncocytoma, highlighting a pivotal diagnostic crossroads.

**Case presentation:**

A 48-year-old woman presented with a right breast lump. Biopsy confirmed HER2-positive invasive carcinoma. Staging positron emission tomography computed tomography revealed an 18F- fluorodeoxyglucose (FDG)-avid left renal mass, highly suspicious for metastasis. A subsequent renal biopsy revealed an oncocytic neoplasm. This finding prompted a curative-intent strategy: the patient underwent simultaneous right breast-conserving surgery and left laparoscopic partial nephrectomy. Final histopathology confirmed stage pT1N0 breast cancer and renal oncocytoma. The patient recovered well and received adjuvant paclitaxel-based chemotherapy with trastuzumab, followed by radiation to the breast, and is planned for continuation of trastuzumab to complete 1 year of anti-HER2 therapy. At 6 months of follow-up, the patient remains asymptomatic with no clinical or radiological evidence of disease recurrence.

**Discussion:**

This case highlights a critical clinical caveat: benign lesions can masquerade as metastases. Our report describes the management of synchronous HER2-positive breast cancer and renal oncocytoma. It exemplifies how a biopsy-driven approach prevented unnecessary systemic therapy and radical nephrectomy, preserving renal function and upholding curative intent.

**Conclusion:**

This experience mandates that tissue confirmation of synchronous lesions be integral to staging protocols. Multidisciplinary review is indispensable for navigating such complex presentations and avoiding therapeutic misadventures.

## Introduction

Current guidelines recommend the use of positron emission tomography computed tomography (PET-CT) only in the presence of signs and/or clinical symptoms of metastatic disease or in stage IV or recurrent disease in breast cancer. In some instances, it may be considered optional under current guidelines for patients with lymph node involvement or HER2-positive disease. The increasing sensitivity of modern imaging, particularly PET-CT, frequently reveals synchronous lesions in patients with cancer, posing a formidable diagnostic challenge^[^1^]^. The immediate and ominous presumption is often metastasis, which can steer management irrevocably toward palliative intent. Renal oncocytoma, a benign tumor, is an increasing concern for urologists, oncologists, and nephrologists due to its difficult differential diagnosis from renal cell carcinoma (RCC) and its frequent overtreatment. It accounts for 3–7.3% of renal neoplasms and is a radiological mimicker of metastasis^[^2^]^. Its radiological profile – often showing enhancement on CT and, as this case demonstrates, significant 18F- fluorodeoxyglucose (FDG) avidity on PET-CT – can be indistinguishable from RCC or metastatic deposits^[^3^]^. Synchronous malignancies involving the breast and kidney are sporadic and have been reported in the literature mainly as breast cancer occurring with RCC rather than benign renal tumors. Two case reports of synchronous breast cancer and renal malignancy highlight the diagnostic and therapeutic complexity of dual primary tumors^[^4,5^]^. We report a clinically pivotal case of a woman with early-stage, HER2-positive breast carcinoma and a contralateral renal mass initially deemed metastatic. The resolution of this diagnostic dilemma through meticulous pathological correlation and a multidisciplinary approach underscores a vital oncologic principle: radiographic suspicion must be validated by histologic proof^[^6^]^. Unlike previously reported cases, the present report describes synchronous HER2-positive breast cancer and renal oncocytoma managed through a biopsy-confirmed, single-stage curative surgical approach. To our knowledge, this is the first report detailing the successful single-stage surgical management of synchronous HER2-positive breast cancer and renal oncocytoma. This case report has been reported in line with the SCARE 2025 criteria^[^7^]^.

HIGHLIGHTS

- Benign renal oncocytomas can exhibit significant FDG avidity on positron emission tomography computed tomography, mimicking metastatic disease in patients with cancer.
- Histopathological verification of synchronous lesions is non-negotiable before committing to a palliative treatment pathway.
- A multidisciplinary tumor board is crucial for interpreting discordant findings and planning integrated, organ-preserving surgeries.
- Simultaneous minimally invasive resection of synchronous primary tumors is a feasible and safe approach in selected patients, optimizing outcomes and resource utilization.

## Case presentation

**Figure 1.**
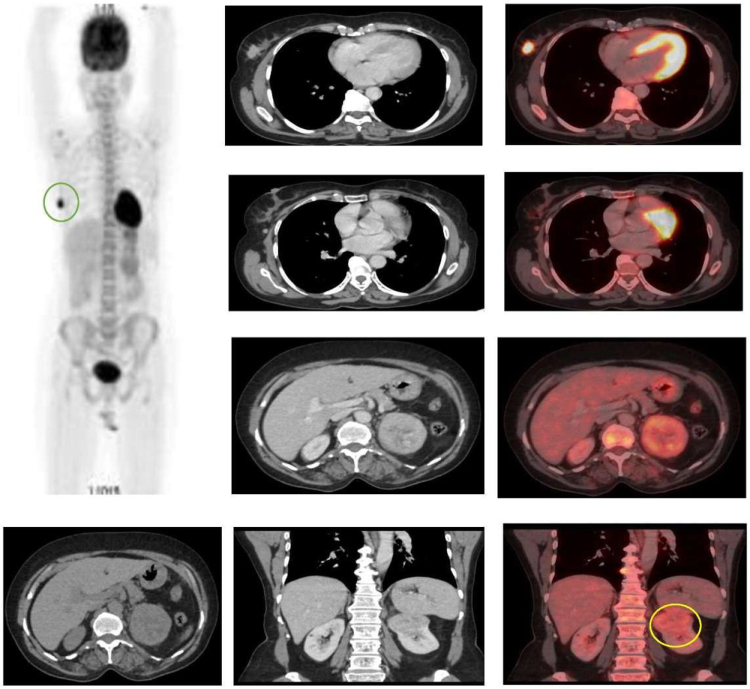
A PET-CT image.

A 48-year-old, premenopausal woman with no significant family history presented with a 6-month history of a palpable lump in her right breast. Clinical examination revealed a 2 × 1 cm firm, mobile mass in the upper outer quadrant with no associated skin changes or palpable axillary lymphadenopathy. A core needle biopsy was performed, which established the diagnosis of invasive carcinoma of no special type, which was estrogen receptor-negative, progesterone receptor-negative, and HER2-positive. As part of the standard staging workup, a whole-body PET-CT scan was obtained. This confirmed a hypermetabolic right breast lesion (Standardized uptake value (SUV) max 15.4) but also disclosed an unexpected, well-circumscribed, heterogeneously enhancing 5.5 cm mass in the upper pole of the left kidney. Crucially, this renal lesion demonstrated moderate FDG avidity (SUV max 5.3), raising a high index of suspicion for a metastatic deposit (Fig. 1).

This finding placed the patient at a therapeutic crossroads. Was this stage IV breast cancer or a concurrent independent pathology? The case was referred to the institutional multidisciplinary tumor board. Contrary to the initial radiographic impression, the board recommended a percutaneous renal mass biopsy to guide further management. Histopathological examination of the renal biopsy specimen revealed a population of oncocytic cells with eosinophilic, granular cytoplasm, arranged in nests, and lacking significant nuclear atypia or mitotic activity – features highly suggestive of a renal oncocytoma.

**Figure 2.**
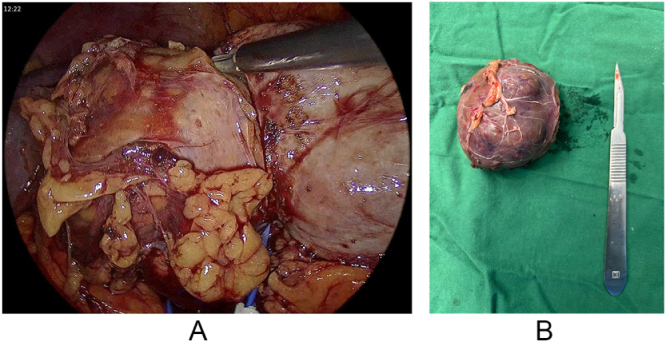
(a) Laparoscopic left partial nephrectomy. (b) Postoperative left partial nephrectomy specimen.

**Figure 3.**
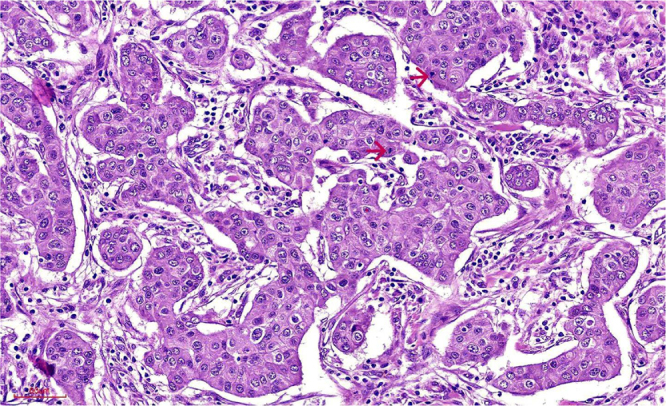
Tumor cells have large, round nuclei, moderate-to-marked nuclear atypia, vesicular chromatin, conspicuous nucleoli, and eosinophilic cytoplasm. Mitotic activity was frequent.

**Figure 4.**
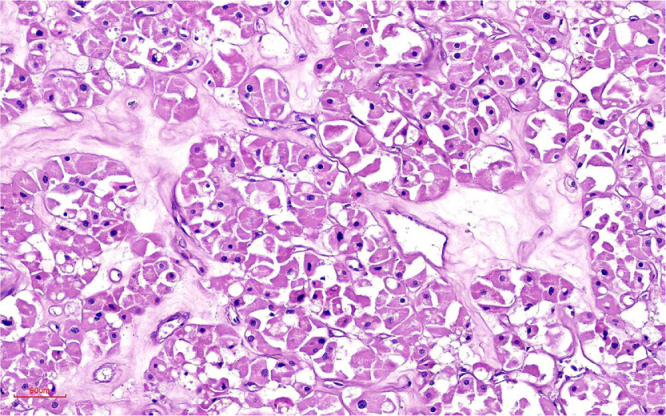
Tumor cells have voluminous eosinophilic granular cytoplasm, a distinct cytoplasmic border, and round pyknotic nuclei (H&E, 200×).

Armed with this definitive diagnosis, the treatment plan was radically altered. Instead of palliative systemic therapy, a dual curative-intent surgical approach was planned. The patient successfully underwent a single anesthetic session comprising a right breast-conserving surgery with sentinel lymph node biopsy and a left laparoscopic partial nephrectomy (Fig. 2a and 2b). The final histopathology report confirmed the initial findings: the breast specimen showed a Grade-3 invasive carcinoma with clear margins, and all 18 axillary lymph nodes were negative for metastasis (stage pT1cN0M0) (Fig. 3). The renal resection specimen exhibited the characteristic architecture and cytology of a renal oncocytoma, and the lesion was completely excised with negative surgical margins (Fig. 4). The patient’s postoperative course was uneventful. She was subsequently initiated on adjuvant systemic therapy consisting of weekly paclitaxel (80 mg/m^2^) in combination with trastuzumab (loading dose 4 mg/kg), planned for 12 cycles, followed by maintenance trastuzumab administered every 3 weeks (6 mg/kg) to complete 1 year of anti-HER2 therapy. Adjuvant radiotherapy to the conserved breast was delivered following completion of chemotherapy and before starting maintenance trastuzumab therapy. Renal function was preserved following partial nephrectomy. Preoperative serum creatinine was 0.8 mg/dL [estimated glomerular filtration rate (eGFR) 90 mL/min/1.73 m^2^], and at 6-week postoperative follow-up, serum creatinine was 0.9 mg/dL (eGFR 85 mL/min/1.73 m^2^), confirming maintained renal function. At 6 months of follow-up, the patient remains asymptomatic with no clinical or radiological evidence of disease recurrence.

## Discussion

Incidentally discovered solid renal masses can have benign or malignant etiologies. Benign entities include oncocytomas, angiomyolipomas, and, rarely, metanephric adenomas and leiomyomas. If there is a history of a known extrarenal primary malignancy, both solid benign renal masses and RCCs should still be considered as possibilities in addition to metastatic disease. This case serves as a paradigm for a critical diagnostic pitfall in modern oncology. The incidental discovery of a synchronous renal mass in a patient with a known extrarenal malignancy is not an automatic death sentence; it is a mandate for pathological confirmation^[^6,8^]^. Renal oncocytoma, while benign, is a notorious radiologic mimicker^[^2^]^. Renal oncocytomas exhibit variable FDG uptake; although often considered PET-negative, several FDG-avid oncocytomas have been reported in the literature. Its potential for FDG uptake, as vividly demonstrated here, adds another layer of complexity and may lead to misclassification of a curable condition as incurable metastatic disease^[^9^]^. Renal oncocytoma is a well-recognized radiologic mimicker of malignant renal tumors and poses a significant diagnostic challenge, particularly in patients with a known extrarenal malignancy. Although renal oncocytomas have traditionally been considered non–FDG-avid, increasing evidence demonstrates that FDG uptake can be variable, with several reports describing moderate to high tracer accumulation, thereby limiting the specificity of PET-CT in differentiating benign from malignant renal lesions. Conventional cross-sectional imaging features, such as a well-circumscribed mass, homogeneous enhancement, and a central stellate scar, may suggest oncocytoma; however, these findings are neither sensitive nor specific and can overlap substantially with those of RCC.

In this context, percutaneous renal mass biopsy plays a critical role in establishing tissue diagnosis and preventing overtreatment. While biopsy has limitations, including sampling error and difficulty distinguishing oncocytoma from chromophobe RCC in select cases, it demonstrates high diagnostic accuracy when oncocytic features are identified and is particularly valuable when biopsy results could significantly alter management. In the present case, biopsy confirmation was pivotal in redirecting treatment from presumed metastatic disease to a curative-intent strategy.

Comparison with previously published studies further clarifies the clinical significance of the present case. Rezzoug *et al* and Üreyen *et al*, in their case reports, described synchronous breast cancer with RCC, in which the renal lesion demonstrates a second malignant primary and required radical surgical management^[^4,5^]^. In contrast, the renal mass in our patient was benign, despite demonstrating FDG uptake on PET-CT. This finding is consistent with observations by Smith *et al*, who reported that renal oncocytomas may show moderate-to-high FDG avidity, thereby closely mimicking malignant renal tumors^[^9^]^.

The decision to pursue simultaneous versus staged surgical management should be individualized and guided by multidisciplinary discussion, taking into account tumor biology, disease stage, surgical complexity, patient performance status, and anticipated recovery. Simultaneous resection may be considered when both lesions are resectable with an acceptable operative risk and when a combined approach minimizes treatment delay and cumulative morbidity. In our patient, early-stage breast cancer amenable to breast-conserving surgery and a localized renal mass suitable for laparoscopic partial nephrectomy favored a single-stage, organ-preserving surgical strategy.

The key decision that altered this patient’s trajectory was the multidisciplinary team’s insistence on a renal biopsy. This intervention de-escalated treatment, preventing an unnecessary radical nephrectomy and, more importantly, the inappropriate institution of lifelong palliative therapy for a patient who was, in fact, curable. Secondary primary malignancies are typically detected incidentally during preoperative screening for distant metastases, raising the question of whether treatment should be administered at different times or simultaneously, and, if at other times, which tumor should be addressed first. The ideal approach should be simultaneous resection of both tumors. The successful execution of simultaneous breast-conserving surgery and laparoscopic partial nephrectomy highlights a sophisticated, patient-centric approach that combines curative intent with organ and function preservation^[^5^]^. This strategy minimized the patient’s physical and psychological burden by consolidating treatment into a single hospitalization and recovery period.

**Figure 5.**
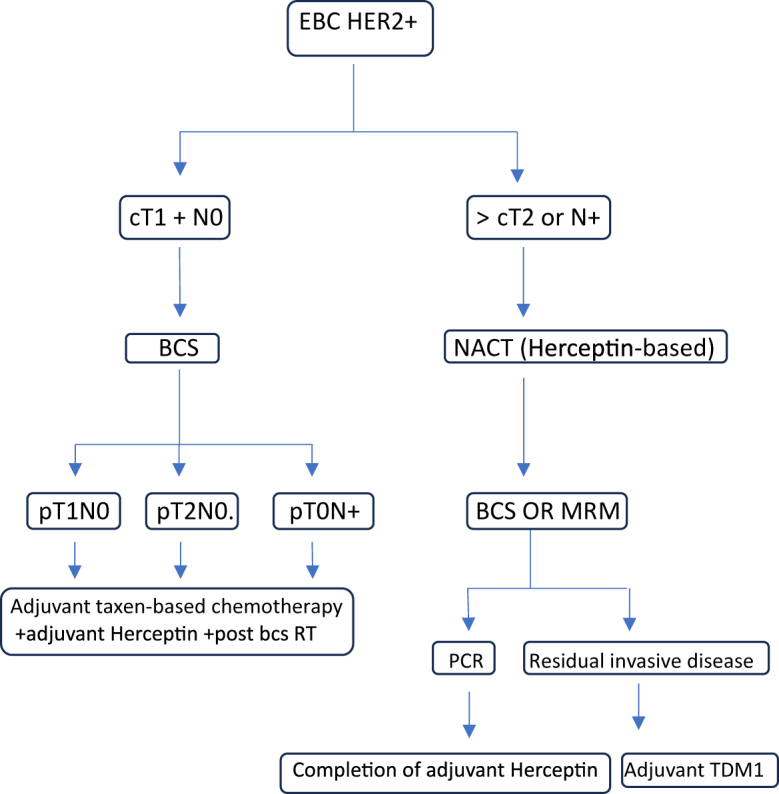
Treatment algorithm for early-stage HER2-positive breast cancer. Adapted from the ESMO Clinical Practice Guidelines and contemporary consensus recommendations^[^11^]^.

While synchronous primary cancers are documented, the specific association of HER2-positive breast cancer with renal oncocytoma is infrequent. However, a woman with breast cancer has a 17% chance of developing a second primary malignancy^[^4^]^. Early-stage HER2-positive breast cancer can directly proceed to surgery without the need for neoadjuvant chemotherapy (Fig. 5)^[^10,11^]^. This report contributes a unique management lesson to the literature. It reinforces that, in the era of advanced imaging, the clinician’s wisdom lies in understanding the limitations of these technologies. The “biopsy-first” principle for solitary synchronous lesions can safeguard against profound management error.

## Conclusion

The adage “trust, but verify” is profoundly applicable in oncology. This case conclusively demonstrates that not all PET-avid renal lesions in a patient with cancer are metastases. Renal oncocytoma must be considered in the differential diagnosis to avoid catastrophic therapeutic missteps. Histopathological confirmation remains the gold standard, and a multidisciplinary team approach is the cornerstone of navigating such complex presentations, ensuring that patients receive accurate staging and optimal, personalized treatment.

## Data Availability

All relevant data supporting the findings of this study are included within the article.

## Patient’s perspective

The patient expressed satisfaction with the combined surgical approach, emphasizing the benefit of single-session treatment and rapid recovery.
